# Temperature effects on TMS-Derived measures of cortical excitability: a systematic review and implications for a warming climate

**DOI:** 10.1016/j.cnp.2026.05.010

**Published:** 2026-06-03

**Authors:** Giulio Peroni, Charlotte Ravenscroft, Simona Balestrini, Sanjay M. Sisodiya

**Affiliations:** a Neuroscience and Human Genetics Department, Meyer Children's Hospital IRCSS, Florence, Italy; b Research Department of Epilepsy, UCL Queen Square Institute of Neurology, London, UK; c Chalfont Centre for Epilepsy, Chalfont St Peter, UK; d Department of Neuroscience, Pharmacology and Child Health, University of Florence, Italy

**Keywords:** Transcranial magnetic stimulation, Temperature, Cortical excitability, Climate change, Heat exposure, Thermoregulation

## Abstract

**Objective:**

To systematically review evidence on the effects of temperature manipulation on transcranial magnetic stimulation (TMS)-derived measures of cortical excitability in healthy individuals and temperature-sensitive neurological conditions.

**Methods:**

Following PRISMA guidelines, we searched PubMed, Cochrane Library, EMBASE, and MEDLINE through November–December 2025. Studies reporting quantitative TMS-EMG or TMS-EEG outcomes after experimentally induced, non-noxious temperature changes were included. Parallel searches examined healthy populations and six temperature-sensitive neurological conditions.

**Results:**

Twenty-one studies in healthy individuals (*n* = 360, mean age 30.9 ± 5.7 years) and one study in multiple sclerosis (MS) met inclusion criteria. Temperature manipulations included whole-body (core changes −2.4 °C to +1.5 °C) and focal interventions (−22 °C to +18 °C). Most studies reported no significant effects on motor evoked potential (MEP) amplitude (73.3%), latency (71.4%), cortical silent period (85.7%), or paired-pulse measures (100%). Effect directions were inconsistent in studies reporting significant findings. The single MS study demonstrated heat-induced impairment, with increased motor threshold and decreased MEP amplitude, not observed in controls.

**Conclusions:**

Experimental TMS studies largely show null effects of temperature on cortical excitability in healthy individuals, contrasting with epidemiological evidence of temperature-related neurological vulnerability. This discrepancy likely reflects modest brain temperature changes, peripheral confounds, and limited ecological validity of current paradigms.

**Significance:**

There is a critical need for climate-relevant experimental designs incorporating sustained thermal exposure and direct cortical readouts (e.g., TMS-EEG) in temperature-sensitive populations to elucidate mechanisms of thermal vulnerability.

## Highlights

• TMS studies show mostly null effects of temperature on cortical excitability.
• Small core temperature changes may not affect excitability in healthy brains.
• Climate-relevant designs and TMS-EEG are needed for future research.

## 1 Introduction

There is an urgent need for neurological approaches to better understand the effects of climate change on the human brain. Since the beginning of the industrial revolution, the mean temperature of the earth has increased by 1.5 °C (Goessling et al., 2025), leading to more frequent and intense extreme weather events globally. Temperature strongly influences biochemical reaction rates and ion channel function, and mounting evidence shows how several neurological conditions are particularly sensitive to environmental modulators (Sisodiya et al., 2024), including temperature changes.

Among the best characterized neurological conditions sensitive to temperature are genetic epilepsy with febrile seizures plus (GEFS+) and Dravet syndrome (DS), which show marked susceptibility to even modest elevations in body temperature and rapid changes in ambient temperature, typically resulting in seizures during febrile episodes in childhood (Scheffer and Nabbout, 2019). Beyond genetic channelopathies, environmental heat exposure has also been increasingly recognized as a precipitant in broader epilepsy populations (McNicholas et al., 2024).

Heat-induced symptom exacerbation is equally well established in multiple sclerosis (MS) and other demyelinating diseases (Christogianni et al., 2018), with transient heat-induced worsening of symptoms documented as early as 1890 (later known as Uhthoff's Phenomenon) and temperature-related increases in emergency department visits reported (Byun et al., 2020).

A similar increase in emergency department visits has been found for migraine during heat waves (Yilmaz et al., 2015), although a recent scoping review (Kelbert and Tobin, 2025) showed conflicting data, suggesting that temperature effects may vary by regional climate, with cold exposure in colder regions potentially being a more significant trigger than heat.

In addition, both increases and decreases in ambient temperature seem to affect stroke incidence. A large US cohort was found to have increased odds of stroke hospitalization with larger daily temperature fluctuations, whereas in the humid continental climate of the Northeast US, higher average annual temperatures were associated with decreased hospitalizations (Lichtman et al., 2016). Conversely, European studies point to an increased stroke incidence with warmer summer temperatures (De Bont et al., 2023), and high ambient temperatures contribute substantially to global stroke burden, accounting for 48,000 deaths and 1.01 million disability-adjusted life years in 2019, with increasing trends from 1990 to 2019 (Bo et al., 2023). These findings suggest that populations in climates characterized by temperature extremes, whether hot or cold, may experience distinct physiological vulnerabilities to their predominant climatic stressor. These vulnerabilities may be exacerbated by anthropogenic climate change, as rising global temperatures and increasing frequency of extreme events may disproportionately affect people with neurological conditions, especially in low and middle-income countries (Sisodiya, 2023).

Beyond direct neurophysiological effects, elevated ambient temperatures increase neurological symptom burden through aggravation of established precipitants and physiological stressors. Heat exposure degrades sleep quality and duration, leading to insufficient sleep across ages (Obradovich et al., 2017), worsening symptoms or outcomes in neurological conditions including epilepsy (Díaz-Negrillo, 2013), migraine (Lucchesi et al., 2016), and stroke (Khot and Morgenstern, 2019). Heat-induced dehydration further increases physiological stress by unbalancing electrolyte homeostasis, thereby altering neuronal membrane excitability even in people without epilepsy (Nardone et al., 2016). Furthermore, chronic heat stress elevates psychological stress and fatigue levels, which themselves constitute symptom precipitants (Ferlisi and Shorvon, 2014; Lucchesi et al., 2016). This cascade of temperature-mediated physiological alterations may synergistically lower thresholds for symptom expression.

Transcranial magnetic stimulation (TMS) paired with neurophysiological readouts such as electroencephalography (EEG) or electromyography (EMG) actively probes cortical circuits. TMS can be used to provide an overall measure of cortical reactivity, determined by the balance of excitatory and inhibitory mechanisms within cortical and corticospinal circuits. Understanding how temperature changes affect TMS-induced cortical responses could elucidate mechanisms underlying heat-related neurologic vulnerability in both healthy individuals and those with neurological conditions.

In this context, TMS offers a mechanistic approach to quantify cortical reactivity and intracortical inhibition/facilitation under controlled physiological perturbations. Here we systematically reviewed human studies that experimentally manipulated temperature and measured quantitative TMS outcomes, both in healthy participants and in temperature-sensitive neurological conditions. Our objectives were to (i) determine whether temperature changes measurably alter TMS-derived markers of cortical reactivity, and (ii) identify methodological features that may explain heterogeneity across findings.

## 2 Methods

### 2.1 Search procedure

We conducted this systematic review as part of the registered review CRD42024521804 following PRISMA reporting guidelines (Page et al., 2021). We performed an initial structured search of PubMed, the Cochrane Library, EMBASE, and MEDLINE on 23 November 2025, combining terms associated with TMS, temperature, and cortical reactivity, to gather data on the general population. Then, on 9 December 2025, we performed six more structured searches on the same databases adding six neurological conditions known to be temperature-sensitive to the search string (epilepsy, MS, migraine, neuropathy, stroke, periodic paralysis) to explore impacts of temperature changes on clinical populations (for the full list of search terms used, please see Supplementary Materials S1). A primary selection of articles for eligibility was conducted by two authors (CR, GP) based on titles and abstracts. Discrepancies were resolved by consensus or by a third author (SB). Full-text articles were screened for inclusion criteria. Reference lists and articles citing the inclusions identified using Google Scholar were also screened to find additional articles.

### 2.2 Inclusion/exclusion criteria

The following inclusion criteria were applied:

1) Research performed in humans
2) Original research
3) Peer-reviewed
4) Reports a quantitative analysis of EEG or EMG responses to single or paired pulse TMS
5) Investigates the effect of temperature changes through direct temperature manipulation
6) Reports a quantifiable temperature change or temperature reached

For condition-specific reviews (epilepsy, MS, migraine, periodic paralysis, neuropathy, and stroke), one additional criterion was applied: research performed in individuals diagnosed with the respective condition.

For the general population review, no specific health condition criterion was required, though participants were expected to be free from neurological disorders.

The following exclusion criteria were applied to all articles:

1) Reports exclusively TMS measures of cortical plasticity
2) Reviews, abstracts, and conference articles
3) Reports data from temperature variations leading to painful stimulation (unless a non-painful comparison was included)

### 2.3 Data extraction and reporting of findings

For each included study, we recorded the impact of temperature on reported TMS measures of cortical reactivity. Given the anticipated differences in study design and TMS protocols, we did not perform quantitative data synthesis.

### 2.4 Quality reporting

Each included article was independently scored by two authors (CR, GP) for methodological quality based on an international consensus checklist (Chipchase et al., 2012). For TMS-EEG studies, relevant methodological parameters were noted.

## 3 Results

**Fig. 1:**
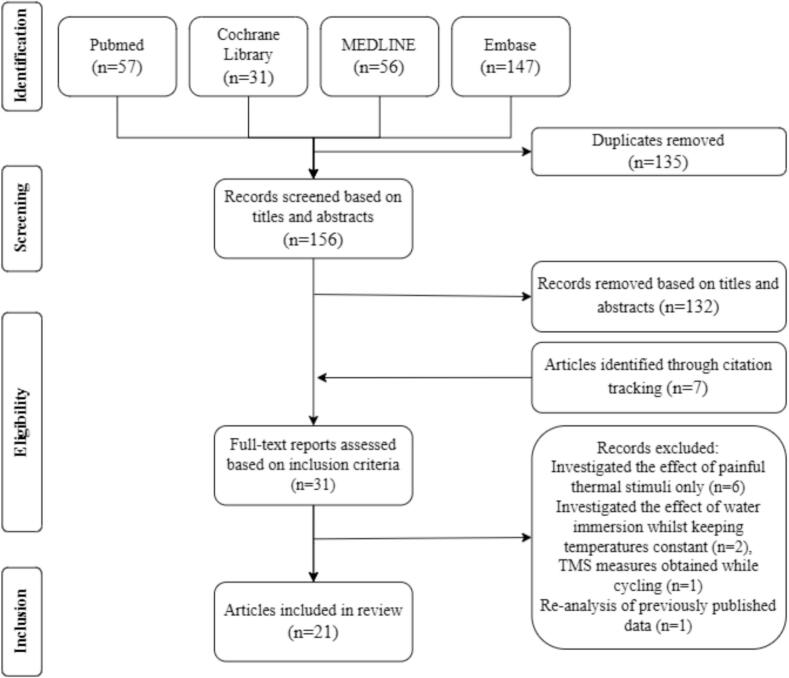
Study design.

Seven parallel systematic literature reviews were conducted to examine the effects of temperature manipulation on TMS measures: one investigating the general population, and the others focused on individuals with epilepsy, MS, migraine, neuropathy, stroke, and periodic paralysis Fig. 1.

For the general population review, the initial search yielded 291 records: 57 from PubMed, 31 from Cochrane Library, 56 from MEDLINE, and 147 from Embase. Citation tracking from the clinical populations' reviews added 7 more records. Following removal of 135 duplicates, 156 unique records underwent title and abstract screening. Of these, 132 were excluded as not meeting inclusion criteria, leaving 24 articles for full-text review. An additional 7 studies were identified through citation tracking of included articles and relevant reviews, and 5 were added from cross-referencing with condition-specific reviews. During full-text assessment, 15 studies were excluded for the following reasons: nine focused exclusively on painful thermal stimulation without temperature change measurement, two involved water immersion protocols maintaining constant body temperature, one examined TMS exclusively during cycling exercise, one offered no comparison between baseline and painless heat conditions, and one represented a reanalysis of previously published data. This process resulted in 21 studies included in the final review.

For the condition-specific reviews, PRISMA charts on records identified, duplicates removed, and exclusions at each screening stage are provided in Supplementary Materials S2. Across all six reviews, only one study, investigating MS, met inclusion criteria and was therefore included in this review.

### 3.1 Study characteristics

The quality of reporting across studies varied significantly, particularly concerning technical parameters. Table 1 illustrates single pulse TMS-EMG parameters, while a comprehensive evaluation including paired pulse TMS-EMG and TMS-EEG is available in supplementary S3.

**Table 1:** TMS-EMG studies quality checklist (single pulse studies only).

	Todd et al., 2005	Todd et al., 2007	Bender et al., 2011	Cahill et al., 2011	Dubé and Mercier, 2011	Gaoua et al., 2011	Iguchi and Shields, 2011	Ross et al., 2012	White et al., 2013	Tremblay et al., 2015	Littmann and Shields, 2016	Ansari et al., 2018a	Ansari et al., 2018b	Magara et al., 2018	Ansari and Tremblay, 2019	Magara et al., 2021	Hurrie et al., 2022	Molenaar et al., 2022	Chowdhury et al., 2023	Talebian-Nia et al., 2023	De Martino et al., 2023
Age	✓	✓	✓	✓	✓	✓	✓	✓	✓	✓	✓	✓	✓	✓	✓	✓	✓	✓	✓	✓	✓
Sex	✓	✓	✓	✓	✓	✓	✓	✓	✓	✓	✓	✓	✓	✓	✓	✓	✓	✓	✓	✓	✓
Handedness	✓	✓	✓	✕	✓	✕	✓	✕	✓	✓	✓	✓	✓	✕	✓	✕	✓	✓	✕	✓	✕
Medication use	✕	✕	✓	✕	✕	✕	✕	✕	✕	✕	✕	✕	✕	✓	✕	✓	✓	✓	✓	✕	✓
Use of CNS active drugs	✕	✕	✓	✕	✕	✕	✕	✕	✕	✕	✕	✕	✕	✓	✕	✓	✓	✓	✓	✕	✓
Presence of neurological disorders	✕	✕	✓	✓	✕	✕	✓	✕	✓	✕	✓	✕	✓	✓	✓	✓	✓	✓	✓	✓	✓
Presence of psychiatric disorders	✕	✕	✕	✕	✕	✕	✕	✕	✕	✕	✕	✕	✕	✕	✕	✕	✕	✕	✓	✕	✓
Any medical conditions	✕	✕	✓	✓	✓	✕	✕	✕	✕	✕	✕	✕	✓	✓	✕	✓	✓	✕	✓	✕	✓
History of specific repetitive motor activity	✕	✕	✓	✓	✕	✕	✓	✓	✓	✕	✓	✕	✕	✕	✕	✕	✓	✓	✕	✕	✕
Position of EMG electrodes	✓	✓	✓	✓	✓	✓	✓	✓	✓	✓	✓	✓	✓	✓	✓	✓	✓	✓	✓	✓	✓
Relaxation/ contraction of target muscles	✓	✓	✓	✓	✓	✓	✓	✓	✓	✕	✕	✓	✓	✓	✓	✓	✓	✓	✓	✓	✓
Prior motor activity of the tested muscle	✕	✕	✕	✓	✕	✓	✓	✕	✕	✕	✕	✕	✕	✕	✕	✕	✕	✓	✕	✕	✕
Level of relaxation of other muscles	✕	✕	✕	✕	✓	✕	✓	✕	✕	✕	✕	✕	✕	✕	✕	✕	✓	✓	✕	✕	✕
Coil type (size and geometry)	✓	✓	✓	✓	✓	✓	✓	✓	✓	✓	✓	✓	✓	✓	✓	✓	✓	✓	✓	✓	✓
Coil shape	O	O	8	O	8	O	8	O	O	8	8	8	8	8	8	8	O	O	8	O	8
Coil orientation	✕	✕	✓	✕	✕	✕	✓	✕	✕	✕	✓	✕	✕	✕	✓	✕	✓	✓	✓	✕	✓
Direction of induced current	✓	✓	✓	✓	✕	✓	✓	✕	✕	✕	✓	✕	✕	✕	✕	✕	✓	✓	✓	✕	✕
Coil location	✓	✓	✓	✓	✓	✓	✓	✓	✓	✓	✓	✓	✓	✓	✓	✓	✓	✓	✓	✓	✓
Type of stimulator used (e.g. brand)	✓	✓	✓	✓	✓	✓	✓	✓	✓	✓	✓	✓	✓	✓	✓	✓	✓	✓	✓	✓	✓
Stimulation intensity	✓	✓	✓	✓	✓	✓	✓	✓	✓	✓	✓	✓	✓	✓	✓	✓	✓	✓	✓	✓	✓
Pulse shape (monophasic or biphasic)	✕	✕	✕	✕	✕	✕	✓	✕	✕	✕	✕	✕	✕	✕	✕	✕	✕	✕	✓	✕	✓
Determination of optimal hotspot	✕	✕	✓	✓	✓	✕	✓	✓	✓	✓	✓	✓	✓	✓	✓	✓	✕	✕	✓	✕	✓
The time between MEP trials	✓	✓	✕	✓	✓	✕	✓	✕	✓	✕	✕	✓	✓	✕	✕	✕	✓	✕	✓	✓	✓
Time between days of testing	✕	✕	✓	✕	NA	✓	✓	✕	✓	NA	✓	NA	✓	NA	NA	✕	✓	✓	NA	✓	NA
Subject attention during testing	✕	✕	✕	✕	✕	✕	✕	✕	✕	✕	✕	✓	✓	✕	✓	✕	✕	✕	✓	✕	✓
Method for determining threshold (aMT/rMT)	NA	NA	✓	NA	✓	✕	✓	✓	✓	✓	✓	✓	✓	✓	✓	✓	✕	✕	✓	✓	✓
Number of MEP trials	✓	✓	✓	✓	✓	✓	✓	✓	✓	✓	✓	✓	✓	✓	✓	✓	✓	✓	✓	✓	✕
Method for determining MEP size during analysis	✓	✓	✓	✕	✓	✕	✓	✓	✓	✓	✓	✓	✓	✓	✓	✓	✓	✕	✓	✓	✕
Size of unconditioned MEP	✓	✓	✓	✓	✕	✕	✕	✓	✓	✕	✕	✕	✕	✕	✓	✕	✕	✕	✕	✕	✕
Neuronavigation used	✕	✕	✕	✕	✓	✕	✕	✕	✕	✕	✓	✕	✕	✕	✕	✕	✕	✕	✕	✕	✓

All 21 selected papers provided basic demographic data, reporting age and sex, while two thirds (*n* = 14, 66.7%) indicated handedness and presence of neurological disorders (regardless of study aim). Less than half reported screening against TMS contraindications (*n* = 10, 47.6%) or any other medical condition (*n* = 9, 42.9%), while only a third reported on use of drugs active on the central nervous system (CNS) (*n* = 7, 33.3%) and even fewer mentioned screening for psychiatric conditions (*n* = 2, 9.5%).

Substantial variability was present in TMS protocols and outcome measures across the included studies. Nineteen studies recorded TMS responses via EMG (TMS-EMG), with seventeen utilizing single-pulse TMS and two employing paired-pulse TMS (ppTMS). Two studies coupled TMS with EEG (TMS-EEG), one of which also included EMG.

The basic electrophysiological setup was consistently described: target muscles, coil type, brand and model, stimulation location and intensity were always reported. Target muscle activation status (*n* = 19, 90.5%) and number of motor evoked potentials (MEPs) recorded (*n* = 20, 95.2%) were also widely reported. Beyond these data, only around two thirds described their method to determine hotspot (*n* = 15, 71.4%), threshold (active or resting) (*n* = 14, 66.7%), and time between TMS trials (*n* = 12, 57.1%). Less than half reported impactful stimulation parameters as coil orientation (*n* = 9, 42.9%), direction of induced current (antero-posterior or postero-anterior) (*n* = 10, 47.6%). Reporting was poorer for more nuanced details such as prior motor activity of the tested muscle (*n* = 4, 19%) and of other muscles (n = 4, 19%), the subject's arousal level (*n* = 6, 28.6%), and pulse shape (*n* = 3, 14.3%) (although this could be derived in most cases from the stimulator model used). In a subset (n = 14) of studies stimulations happened over two days to either compare different conditions (Iguchi and Shields, 2011), verify the reproducibility of results for the same condition (Todd et al., 2007), or to familiarize individuals with the study protocol in a first session (Talebian-Nia et al., 2023). Of these 14, 9 (64.3%) clearly stated how many days passed between sessions. The number of stimulation trials ranged in TMS-EMG studies from 3(Todd et al., 2005) to 66 (Chowdhury et al., 2023) pulses per condition, while in TMS-EEG studies ranged from 66 (Chowdhury et al., 2023) to 180 (De Martino et al., 2023).

### 3.2 Subjects

The 21 included studies enrolled a total of 371 individuals, of which 159 (42.9%) were females. The gender imbalance may be due to some studies that either only included (Tremblay et al., 2015) or heavily skewed toward males (Cahill et al., 2011; Hurrie et al., 2022).All but one study aimed to explore variation in cortical excitability between different thermal conditions, and therefore mostly recruited healthy individuals around their mid-twenties to early thirties (31.1 ± 5.7 years old across studies). While “healthy” individuals were the main population tested, application of standard TMS exclusion criteria was reported in only 9 (42.9%) sources. Screening for CNS-active drug use and psychiatric disorders was reported in 7 (33.3%) and 2 (9.5%) cases respectively. In two studies investigating focal distal cooling, individuals were screened for acute or chronic musculoskeletal or neurological conditions that might be exacerbated by cold exposure (Ansari et al., 2018a). Only one study investigated a clinical population, (White et al., 2013), which included 11 people with MS (definite relapsing-remitting MS), matched to 11 healthy individuals.

### 3.3 Temperature paradigms

There was considerable heterogeneity in the methods used to induce thermal changes across studies. Temperature manipulations involved the whole body in 11 studies (52.4%) and focal regions in 10 (47.6%). Heating was applied in 13 interventions (61.9%), cooling in 3 (14.3%), and both in 5 (23.8%). Focal interventions targeted various upper extremity sites including the forearm (De Martino et al., 2023), hand (Dubé and Mercier, 2011), all fingers (Ansari et al., 2018a), index finger (Ansari et al., 2018b), as well as scalp (Tremblay et al., 2015) and tongue (Magara et al., 2018) Fig. 2.

**Fig. 2:**
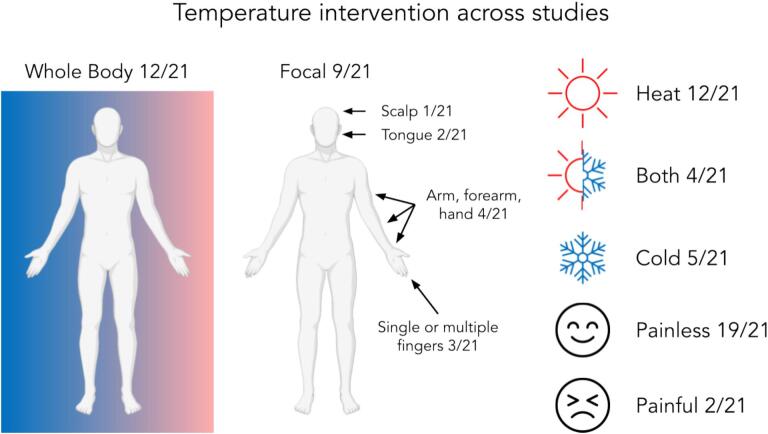
Temperature interventions across studies.

Studies investigating whole-body temperature changes achieved deep temperature variations ranging from approximately −2.4 °C[26] to +1.5 °C (Gaoua et al., 2011), measured via oesophageal (Cahill et al., 2011), rectal (Ross et al., 2012), tympanic (Littmann and Shields, 2016), or intestinal routes (Gaoua et al., 2011).

In contrast, focal interventions showed larger temperature deltas, ranging from skin temperature reductions of −12.5 °C (Tremblay et al., 2015) to increases reaching painful thresholds of 46–50 °C (Chowdhury et al., 2023; Dubé and Mercier, 2011). These differences in intervention type (whole-body vs focal; painful vs non-painful) limit comparability, as these approaches likely engage distinct physiological mechanisms.

### 3.4 TMS-EMG results

MEPs were predominantly recorded from upper limb muscles: first dorsal interosseus in 6 studies (28.6%), biceps brachii and related muscles in 6 studies (28.6%), abductor pollicis brevis (APB) in 3 studies (14.3%), and abductor pollicis longus, opponens pollicis, extensor carpi radialis brevis, flexor carpi radialis, vastus lateralis, deep finger flexors, and knee extensors in 1 study (4.8%) each. Two studies (Magara et al., 2021; Magara et al., 2018) explored motor pathways involved in human swallowing, with EMG recorded from pharyngeal muscles and ABP. EMG measurements were conducted under various muscle contraction states (rest, sustained maximal voluntary contractions, and submaximal contractions).

The dependent variables examined were similarly diverse, encompassing MEP amplitude, MEP latency, cortical silent period (CSP), short-interval intracortical inhibition (SICI), long-interval intracortical inhibition (LICI), intracortical facilitation (ICF), and TMS-evoked potential (TEP) component amplitude and latency. This methodological heterogeneity substantially limits direct comparisons between studies and complicates efforts to synthesize conclusions regarding temperature effects on cortical excitability.

MEP amplitude, considered an index of overall corticospinal excitability, was the most frequently reported outcome measure, assessed in 15 of the 21 studies (71.4%). The predominant finding was no significant change in MEP amplitude following temperature manipulation. Of the 15 studies reporting MEP amplitude, 11 studies (73.3%) found no significant alterations in at least their primary comparison, with several studies explicitly reporting no statistically significant differences across multiple temperature conditions or time points. Among studies demonstrating significant effects, the direction of change was inconsistent: (Littmann and Shields, 2016) reported increased MEP amplitude following whole-body heating, whereas (Gaoua et al., 2011) found no changes with a whole-body temperature increase of 0.5 °C and a decrease in MEP amplitudes when whole-body temperature was increased by 1 °C and 1.5 °C. (Dubé and Mercier, 2011) found no changes in painless focal heat stimulation, and decreased amplitudes during painful focal heating, while (Tremblay et al., 2015) observed reduced amplitudes following focal cooling of the contralateral hemiscalp. Several investigations reported mixed or variable results, with effects appearing dependent on specific experimental parameters such as distance of the target muscle from the site of thermal stimulation (Magara et al., 2021, Magara et al., 2018), or individual subject characteristics (Ansari et al., 2018a, Ansari et al., 2018b).

Beyond MEP amplitude, other TMS-EMG parameters were examined across studies, with considerably less frequency and similarly inconsistent results. MEP latency, considered an index of corticospinal conduction time, was reported in seven studies, with five demonstrating no significant change following temperature manipulation, one (Cahill et al., 2011) showing increased latency during whole-body hypothermia, and one (White et al., 2013) showing decreased latency after whole-body warming in both healthy individuals and MS patients.

CSP, an indicator of intracortical inhibition mediated primarily through GABA_B_ receptors, was assessed in seven studies. Results were predominantly null, with six studies reporting no significant changes in CSP duration following either heating or cooling interventions. Only one investigation (Molenaar et al., 2022) demonstrated a significant effect, reporting increased CSP duration by 0.38 ± 0.17 ms per degree Celsius of skin temperature reduction during focal arm cooling. Recruitment curves, which describe the relationship between stimulation intensity and MEP amplitude, were examined in only two studies (Littmann and Shields, 2016; White et al., 2013), both of which reported no significant change in the recruitment curve parameters in healthy individuals following whole-body heating.

People with MS, the only clinical sample explored, demonstrated significantly increased resting motor threshold (rMT), decreased MEP amplitude, and reduced recruitment curve slope during whole-body heating (White et al., 2013). An increase in core temperature of 0.6 °C was achieved by warm water passed through a tube-lined perfusion suit, highlighting heat-induced impairment of cortical excitability and illustrating Uhthoff's phenomenon. Additionally, MS patients failed to show the normal heat-induced shortening of central motor conduction time (the time interval between the stimulation of the motor cortex and the arrival of the descending volleys at the spinal level) observed in controls, reflecting the impact of demyelination on temperature-dependent conduction properties.

Paired-pulse TMS measures assessing intracortical circuits were employed only in two studies (Ansari and Tremblay, 2019; Bender et al., 2011) and yielded uniformly null results. Short-interval intracortical inhibition (SICI), reflecting GABA_A_-mediated inhibition, was examined in both studies, reporting no significant changes following temperature manipulation. Long-interval intracortical inhibition (LICI), mediated by GABA_B_ receptors, and intracortical facilitation (ICF), thought to reflect glutamatergic excitatory mechanisms, were assessed only by (Bender et al., 2011), with both measures showing no temperature-related alterations.

### 3.5 TMS-EEG results

TMS-EEG recordings were employed in only two studies (Chowdhury et al., 2023; De Martino et al., 2023), representing a relatively underutilized approach in this field. Both investigations applied focal heat stimulation to the forearm and compared painless to painful warming conditions. The TMS-EEG protocols involved substantially higher trial numbers (66–180 pulses) than TMS-EMG studies, reflecting the increased data requirements for reliable evoked potential analysis. (De Martino et al., 2023) stimulated primary motor cortex (M1) and dorsolateral prefrontal cortex (DLPFC), while (Chowdhury et al., 2023) focused on M1 stimulation. TEP components findings were mixed: (De Martino et al., 2023) reported statistically significant differences in “change from baseline” in amplitudes for P30 (M1) during painful compared to both painless warm stimulation and post-thermal intervention (thermoneutral) and P25 (DLPFC) during painful compared to painless warm stimulation, but no change in P30 latency and both N45 (DLPFC) and N100 (M1) amplitudes and latencies or global mean field power (GMFP) in any condition. Conversely, (Chowdhury et al., 2023) stimulating on M1, found increased N45 and N100 amplitude in frontocentral electrodes and increased P60 amplitude from parieto-occipital electrodes during pain compared to warm conditions. No comparison was undertaken between warm conditions and non-warm baseline.

A summary of results is provided in Table 2, Table 3, a comprehensive format is available in supplementary S4 and S5.

**Table 2:** Results from whole-body temperature intervention studies.

Author	Subjects	Sample (females)	Age (years - mean ± SD)	Stimulation site	Recording site	Induced core temperature change (°C)	TMS-EMG measures									
							MEP amplitude	MEP area	MEP latency	I-O curve	rMT	aMT	CSP	SICI	ICF	LICI
Cooling paradigms																
Cahill et al., 2011	HI	7 (1)	26.4 ± 4	M1	biceps brachii	∼2.4	NC		↑				NC			
Hurrie et al., 2022	HI	12 (4)	26 ± 6	M1	biceps brachii	∼0.6	NC									
Talebian-Nia et al., 2023	HI	8 (4)	26 ± 7	M1	biceps brachii	∼0.8	NC									
Heating paradigms																
Todd et al., 2005	HI	7 (5)	37 ± 11	M1	biceps and triceps brachii	∼1.3		NC					NC			
Todd et al., 2007	HI	7 (5)	36 ± 10	M1	biceps and triceps brachii	∼1.3							NC			
Bender et al., 2011	HI	10 (2)	22 ± 4	M1	flexor carpii radialis	∼1								NC	NC	NC
Gaoua et al., 2011	HI	8 (1)	34 ± 6	M1	APL	∼0.5- ∼ 1.5	NC under 1,5° ↓ above 1,5°						NC			
Iguchi and Shields, 2011	HI	25 (12)	M: 21.2 ± 2.1 F: 23.2 ± 2.2	M1	biceps brachii, brachioradialis, triceps brachii	∼0.82	NC					NC	NC			
Ross et al., 2012	HI	8 (2)	27.2 ± 6.3 y	M1	vastus lateralis	≥2	NC									
White et al., 2013	HI	11 (7)	37.4 ± 9.6	M1	APB	∼0.6	NC		↓	NC	NC		NC			
	MS	11 (7)	38.9 ± 6.9				NC		↓	↓	NC		NC			
Littmann and Shields, 2016	HI	11 (5)	25.2 ± 5.8	M1	FDI	∼2	↑			NC						

APB: Abductor pollicis brevis, APL: Abductor pollicis longus, FDI: first dorsal interosseous, HS: healthy subjects, M1: Primary motor cortex (Brodmann 4), MS: multiple sclerosis patients, NC: no changes.

**Table 3:** Results from focal temperature intervention studies.

Author		Sample (females)	Age	Stimulation site	Recording site	Temperature intervention site	Induced temperature change (°C)	TMS-EMG measures			TMS-EEG measures	
								MEP amplitude	MEP latency	CSP	TEP amplitude	GMFP
Cooling paradigms												
Tremblay et al., 2015		12 (0)	31.5 ± 11.4	M1	FDI	Hemiscalp	10 °C	↓	NC	–	–	–
Ansari et al., 2018b		13 (5)	30 ± 4	M1	Multiple digits	Single digit (SD) Multiple digits (MD)	10.3C (SD) 11.0C (MD)	NC (variable)	NC (variable)	–	–	–
Ansari and Tremblay, 2019		21 (11)	28 ± 7	M1	FDI	Multiple digits	NA	NC (variable)	–	–	–	–
Heating paradigms												
Dubé and Mercier, 2011		15 (7)	27.4 ± 4.9	M1	FDI and opponens pollici	lateral edge of the right hand	painless +4 °C painful +18 °C	NC in heat ↓ in pain	–	–	–	–
De Martino et al., 2023		24 (12)	27 ± 5.5	M1 and DLPFC	scalp EEG	volar region of the right forearm	painless +12 °C painful +17 °C	–	–	–	–	M1: more ↑ in warm than pain DLPFC: NC
Chowdhury et al., 2023	1°exp	29 (11)	26,44 ± 5,5	M1	ECRB	proximal region of the right ECRB	painless +3 ± 1.5 °C painful +14 °C	–	–	–	↑ in pain vs warm	–
	2°exp	10 (6)	26.8 ± 5.9									
	3°exp	10 (6)	28 ± 5.9									
Mixed paradigms												
Ansari et al., 2018a	young	20 (10)	28 ± 5	M1	FDI	index finger	heating +7.4° cooling −8.8∘C	NC (variable)	NC (variable)	–	–	–
	senior	15 (6)	67 ± 4							–	–	–
Magara et al., 2018		18 (5)	27.2 ± 3.5	M1	APB, hypopharynx	tongue	heating +8° cooling −22°	NC (APB) NC (P, hot); ↑ (P, cold)	NC (APB and P)	–	–	–
Magara et al., 2021	1°exp	8 (4)	26.9 ± 2.7	M1	APB, hypopharynx	tongue	heating +8° cooling −22°	NC (APB) NC (P, hot); ↑ (P, cold)	NC (APB and P)	–	–	–
	2°exp	12 (6)	27.2 ± 1.9	M1	APB, hypopharynx					–	–	–
Molenaar et al., 2022		10 (5)	25.7 ± 3.5	M1	finger flexors and extensors	forearm	heating not stated cooling −16.1°	–	–	NC, heat ↑ cold	–	–

APB: Abductor pollicis brevis, DLPFC: Dorsolateral prefrontal cortex (BA9, 46), ERCB: extensor carpi radialis brevis, FDI: first dorsal interosseous, M1: Primary motor cortex (BA4), NC: no changes.

## 4 Discussion

This systematic review showed a discrepancy between epidemiological evidence, reporting clear correlations between temperature changes and neurological symptom burden, and experimental investigations using TMS, that have predominantly failed to detect corresponding alterations in cortical excitability measures even in healthy individuals. Of 21 studies examining temperature effects on TMS parameters in healthy individuals and one study in people with MS(White et al., 2013), the majority reported null findings across MEP amplitude, latency, cortical silent period, and paired-pulse measures. Notably, these studies enrolled predominantly young, male participants, an important limitation given established sex differences exist in thermoregulatory responses to heat stress and across ages (Debray et al., 2025).

The substantial variability in experimental design, temperature manipulation protocols, and TMS methodology significantly limits interpretability and precludes quantitative synthesis. Temperature interventions included whole-body manipulations achieving core temperature changes of −2.4 °C (Cahill et al., 2011) to +1.5 °C (Gaoua et al., 2011), as well as focal applications producing skin temperature alterations from +12 °C (De Martino et al., 2023) to −22 °C (Magara et al., 2021). These approaches are likely affected by different physiological mechanisms: whole-body temperature changes may engage systemic thermoregulatory responses involving metabolic, cardiovascular, and neuroendocrine adjustments, while focal thermal stimulation primarily activates peripheral thermoreceptors with minimal impact on core or brain temperature. Notably, brain temperature also has its own rhythm (Rzechorzek et al., 2022), which wasn't addressed in any study.

Reporting quality varied considerably, particularly regarding technical parameters critical for result interpretation and replication. While basic parameters were consistently documented, fewer than half of studies reported key stimulation parameters such as coil orientation, induced current direction, or screening for CNS-active medications. The predominance of young, healthy males (56.4% male, mean age 28.5 years) limits generalizability to populations most vulnerable to temperature-related symptoms. Mainly, despite well-documented temperature sensitivity of epilepsy, MS, migraine, and stroke, only one study (White et al., 2013) examined a clinical population, showing a marked mismatch between clinical need and the limited availability of protocols that mirror real-world conditions.

Research findings suggest that 20–30 trials are required to obtain reliable measures of MEP amplitude (Biabani et al., 2018; Goldsworthy et al., 2016), and at least 25 trials are required for reliable paired-pulse measures (Biabani et al., 2018). Of the TMS-EMG studies reviewed, only one (Chowdhury et al., 2023) obtained a sufficient number of trials according to these recommendations. The predominant null findings may partly reflect suboptimal TMS stimulation parameters, rather than definitely indicating absence of an effect. A fundamental challenge in interpreting TMS-EMG findings relates to difficulty disentangling cortical from peripheral contributions. Motor evoked potentials reflect integrated output of cortical excitability, corticospinal conduction, spinal motor neuron function, neuromuscular transmission, and muscle contractility, all of which may be temperature-sensitive to varying degrees. The isolated finding of increased MEP latency during whole-body hypothermia (Cahill et al., 2011) likely reflects slowed peripheral nerve conduction rather than altered cortical function, consistent with well-established temperature coefficients (Q₁₀ values) for axonal conduction, though the predominance of null findings for both CSP and MEP latency suggests that temperature changes reached may not substantially affect motor corticospinal conduction time in most experimental contexts, or that these intracortical mechanisms may be relatively resistant to temperature-induced modulation. Similarly, focal limb cooling or heating may alter proprioceptive and cutaneous input, potentially masking direct cortical effects through compensatory modulation. TMS-EEG offers more direct cortical assessment by recording brain activity rather than motor output. However, only two studies employed this approach, both examining primarily painful versus non-painful heat. While these investigations reported some alterations in TEP components between baseline and innocuous heat, the comparison between painful and non-painful thermal stimulation introduces substantial confounds related to pain perception, attention, arousal, and emotional processing rather than temperature alone. The absence of studies comparing TMS-EEG responses during painless temperature manipulations to true thermoneutral baselines represents a significant methodological gap, as current evidence cannot distinguish temperature-specific cortical changes from pain-related modulation of brain state.

A further consideration is the magnitude of temperature change required to measurably alter cortical excitability. Ion channels implicated in neurological disorders, such as the Nav1.1 channels dysfunctional in DS and GEFS+, exhibit Q₁₀ values of 1.2–3, indicating 20–200% kinetic changes per 10 °C (Catterall et al., 2010). However, most whole-body heating studies achieved core temperature increases of only 1–1.5 °C, with brain temperature likely changing less due to cerebral autoregulation. At these modest elevations, ion channel function would change by only 2–5%, potentially insufficient to produce detectable TMS alterations given measurement variability and small sample sizes. However, network nonlinearities could still amplify small kinetic shifts in susceptible brains, especially in channelopathies.

An important limitation of the existing literature is that none of the included studies were designed to investigate climate change impacts on neurological function. Consequently, experimental paradigms prioritized acute, controlled temperature manipulations rather than sustained, ecologically relevant thermal exposures characteristic of real-world heat waves or chronic climate warming. Furthermore, as several included studies did not primarily aim to examine cortical excitability and instead investigated peripheral nerve function or muscle properties (with TMS as an ancillary method) null findings may simply reflect protocols not optimized to detect cortical excitability changes, absence of meaningful brain temperature alteration rather than TMS insensitivity, since focal temperature interventions, while producing larger gradients, primarily affect superficial tissues with minimal core or brain impact.

The discordance between modest laboratory findings and robust epidemiological evidence suggests that single-factor paradigms may be insufficient to capture multifactorial real-world thermal stress. Climate-related health impacts result from complex interactions among physiological stressors accompanying heat: sleep disruption, dehydration, psychological and physical fatigue; their synergistic effects may amplify modest direct temperature effects, producing clinically significant exacerbation despite minimal changes in isolated laboratory manipulations. Nevertheless, understanding cerebral effects of elevated temperature remains important to inform acclimation and protective strategies, especially in people with neurological disorders and potentially compromised thermoregulatory control.

Perhaps most striking is the near-complete absence of TMS studies in populations known to be temperature-sensitive. Despite targeted searches for epilepsy, MS, migraine, neuropathy, stroke, and periodic paralysis, only one MS investigation met inclusion criteria. The sole MS study (White et al., 2013) provides preliminary evidence: while healthy controls showed no recruitment curve changes following whole-body heating, MS patients demonstrated decreased slope, suggesting altered cortical excitability. This aligns with the Uhthoff phenomenon and supports the hypothesis that TMS may detect temperature-related changes in appropriately vulnerable populations.

To develop TMS as a meaningful tool for understanding climate impacts on brain health, future studies must prioritize individuals with temperature-sensitive neurological conditions and include age-stratified designs to account for vulnerable populations. Regarding experimental protocols, whole-body exposure achieving sustained brain temperature variations coupled with deep body measurements, rather than peripheral variations and measurements, may be more representative of real-world temperature change. To better mimic environmental heat, multifactorial paradigms incorporating multiple stressors (controlled sleep restriction, mild dehydration, or psychological stress) may help better understand synergistic effects of real-world heat waves. Moreover, repeated exposure protocols over days or weeks to model sustained heat waves or longitudinal designs tracking TMS measures across seasons could reveal cumulative effects. Lastly, adopting standardized reporting guidelines for TMS-temperature studies and transitioning toward TMS-EEG as primary outcome may provide direct cortical readouts unconfounded by peripheral factors.

## 5 Conclusion

Existing evidence regarding the effect of temperature on TMS measures is characterized by methodological heterogeneity, predominant null findings, and striking absence of research in clinically relevant populations. While epidemiological data demonstrate temperature-related neurological symptom exacerbation, laboratory TMS studies have largely failed to detect corresponding cortical excitability changes. This disconnect may reflect modest brain temperature changes in experimental paradigms, difficulty disentangling cortical effects from peripheral contributions, and failure to model multifactorial real-world thermal stress.

The field faces an important question: is TMS insufficiently sensitive to detect subtle temperature-related cortical changes, or have experimental paradigms been inadequate to produce meaningful brain temperature alterations? This requires studies employing TMS-EEG in temperature-sensitive patient populations, utilizing climate chamber exposures for sustained brain temperature changes, and incorporating concurrent stressors characterizing real heat exposure, within safe, non-noxious ranges and with medical monitoring.

As climate change accelerates and extreme heat events intensify, understanding neurophysiological mechanisms linking temperature to neurological vulnerability becomes increasingly urgent. TMS holds promise for elucidating these mechanisms and identifying at-risk individuals, but realizing this potential requires methodological refinement, focus on vulnerable populations, and frameworks acknowledging the multifactorial nature of climate impacts on brain health.

## CRediT authorship contribution statement

**Giulio Peroni:** Conceptualization, Data curation, Formal analysis, Visualization, Writing – original draft, Writing – review & editing. **Charlotte Ravenscroft:** Conceptualization, Data curation, Writing – original draft, Writing – review & editing. **Simona Balestrini:** Conceptualization, Supervision, Writing – review & editing. **Sanjay M. Sisodiya:** Conceptualization, Supervision, Writing – review & editing.

## Acknowledgements and funding

We are grateful to the Epilepsy Society for their support of this work, and funding to SMS, SB and CR. GP is supported by the Italian 10.13039/501100004726Ministry of Health (EPPERMED2024-329).

## Declaration of competing interest

The authors declare that they have no known competing financial interests or personal relationships that could have appeared to influence the work reported in this paper.
