# Supplementary material for: Temperature effects on TMS-Derived measures of cortical excitability: a systematic review and implications for a warming climate
Source: Clin Neurophysiol Pract. 2026 Jun 3;11:416–25. doi: 10.1016/j.cnp.2026.05.010 (PMC13279741; doi:10.1016/j.cnp.2026.05.010)
Supplement: Supplementary file 2 — Supplementary material 2 [file mmc2.docx]

**Supplementary materials S1 – full search terms**

How does **temperature** affect TMS measures of excitability in **healthy individuals?**

("TMS" OR "Transcranial magnetic stimulation") AND ("temperature" OR "heat" OR "warmth" OR "cold" OR "climate" OR "hyperthermia" OR "hypothermia") AND ("TMS-EMG" OR "TMS-EEG" OR "Motor-evoked potential" OR "MEP" OR "TMS-evoked potential" OR "TEP" OR "SICI" OR "LICI" or "cortical excitability" OR "cortical reactivity")

How does **temperature** affect TMS measures of excitability in **people with epilepsy?**

(“epilepsy”) AND ("TMS" OR "Transcranial magnetic stimulation") AND ("temperature" OR "heat" OR "warmth" OR "cold" OR "climate" OR "hyperthermia" OR "hypothermia") AND ("TMS-EMG" OR "TMS-EEG" OR "Motor-evoked potential" OR "MEP" OR "TMS-evoked potential" OR "TEP" OR "SICI" OR "LICI" or "cortical excitability" OR "cortical reactivity")

How does **temperature** affect TMS measures of excitability in **people with stroke?**

(“stroke”) AND ("TMS" OR "Transcranial magnetic stimulation") AND ("temperature" OR "heat" OR "warmth" OR "cold" OR "climate" OR "hyperthermia" OR "hypothermia") AND ("TMS-EMG" OR "TMS-EEG" OR "Motor-evoked potential" OR "MEP" OR "TMS-evoked potential" OR "TEP" OR "SICI" OR "LICI" or "cortical excitability" OR "cortical reactivity")

How does **temperature** affect TMS measures of excitability in **people with multiple sclerosis?**

(“multiple sclerosis”) AND ("TMS" OR "Transcranial magnetic stimulation") AND ("temperature" OR "heat" OR "warmth" OR "cold" OR "climate" OR "hyperthermia" OR "hypothermia") AND ("TMS-EMG" OR "TMS-EEG" OR "Motor-evoked potential" OR "MEP" OR "TMS-evoked potential" OR "TEP" OR "SICI" OR "LICI" or "cortical excitability" OR "cortical reactivity")

How does **temperature** affect TMS measures of excitability in **people with migraine?**

(“migraine”) AND ("TMS" OR "Transcranial magnetic stimulation") AND ("temperature" OR "heat" OR "warmth" OR "cold" OR "climate" OR "hyperthermia" OR "hypothermia") AND ("TMS-EMG" OR "TMS-EEG" OR "Motor-evoked potential" OR "MEP" OR "TMS-evoked potential" OR "TEP" OR "SICI" OR "LICI" or "cortical excitability" OR "cortical reactivity")

How does **temperature** affect TMS measures of excitability in **people with neuropathy?**

(“neuropathy”) AND ("TMS" OR "Transcranial magnetic stimulation") AND ("temperature" OR "heat" OR "warmth" OR "cold" OR "climate" OR "hyperthermia" OR "hypothermia") AND ("TMS-EMG" OR "TMS-EEG" OR "Motor-evoked potential" OR "MEP" OR "TMS-evoked potential" OR "TEP" OR "SICI" OR "LICI" or "cortical excitability" OR "cortical reactivity")

How does **temperature** affect TMS measures of excitability in **people with periodic paralysis?**

(“periodic paralysis”) AND ("TMS" OR "Transcranial magnetic stimulation") AND ("temperature" OR "heat" OR "warmth" OR "cold" OR "climate" OR "hyperthermia" OR "hypothermia") AND ("TMS-EMG" OR "TMS-EEG" OR "Motor-evoked potential" OR "MEP" OR "TMS-evoked potential" OR "TEP" OR "SICI" OR "LICI" or "cortical excitability" OR "cortical reactivity")

**Supplementary materials S2 – PRISMA flowcharts for condition-specific reviews**

**Epilepsy**


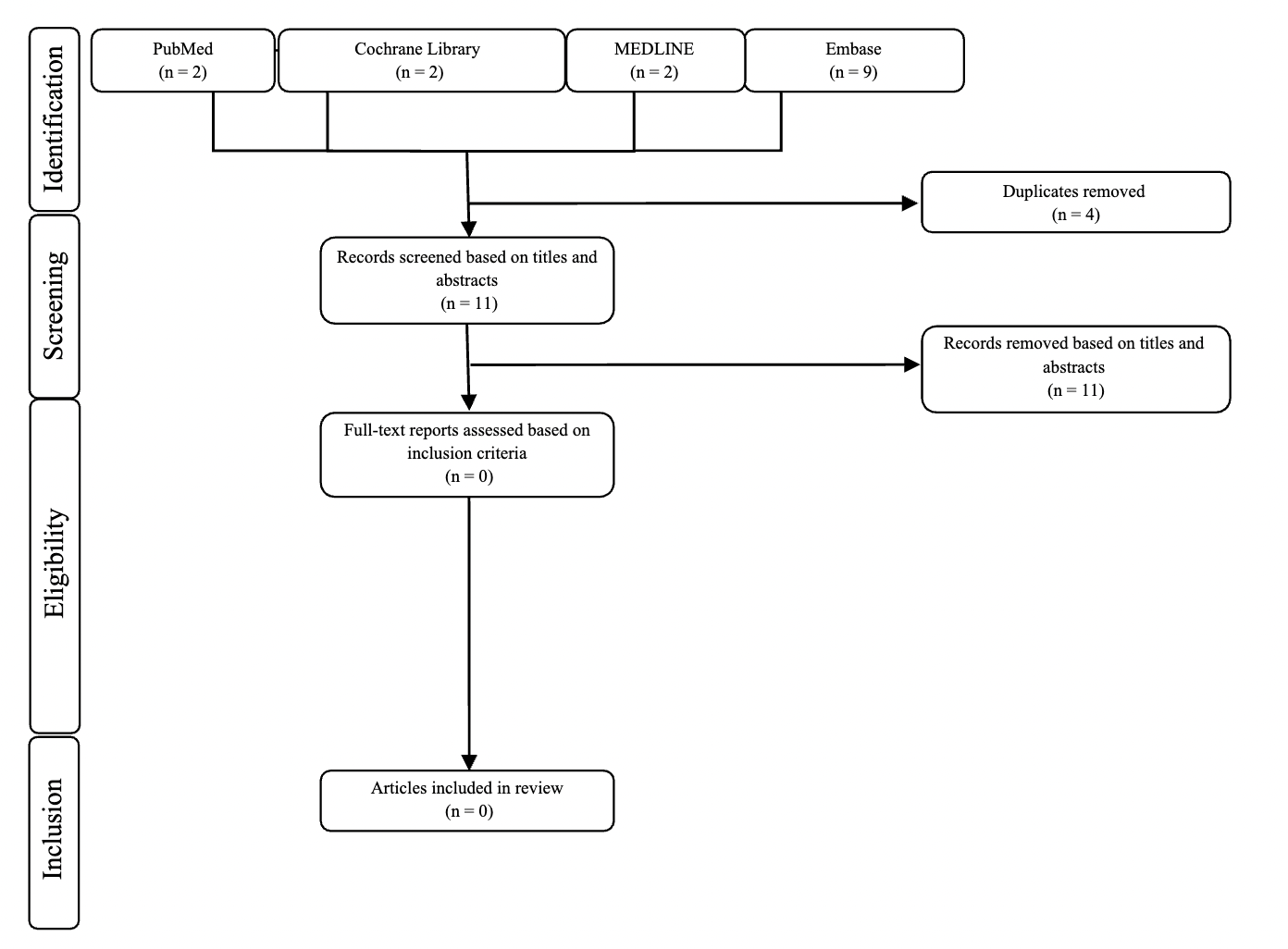


**Multiple Sclerosis**

**
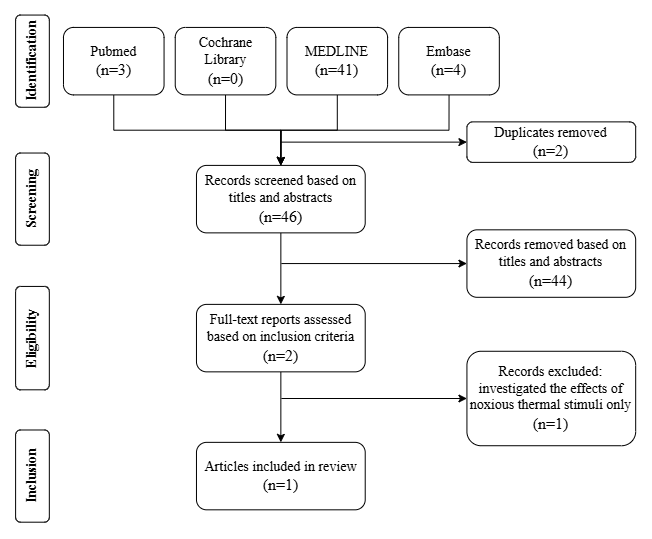
**

**Migraine

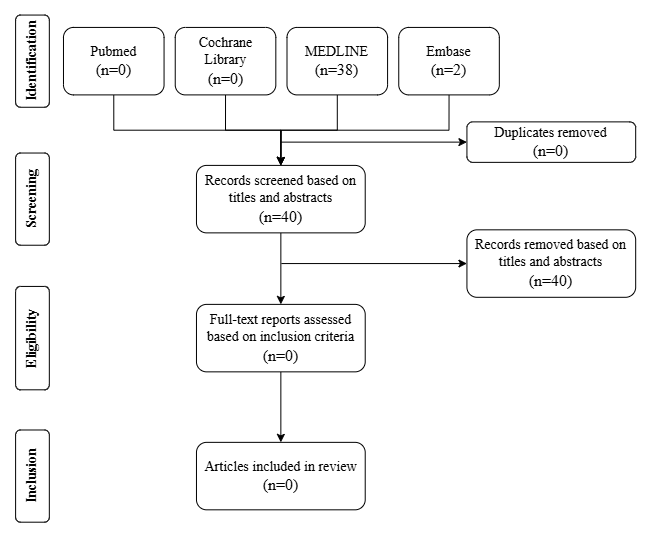
**

**Stroke**

**
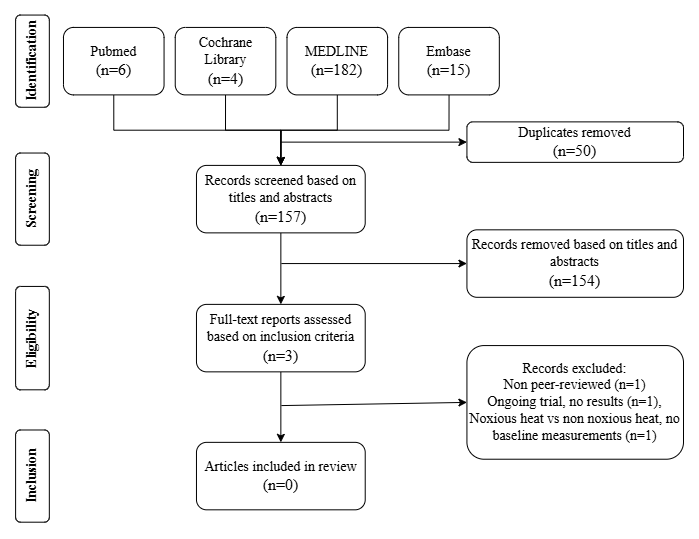
**

**Neuropathy**

**
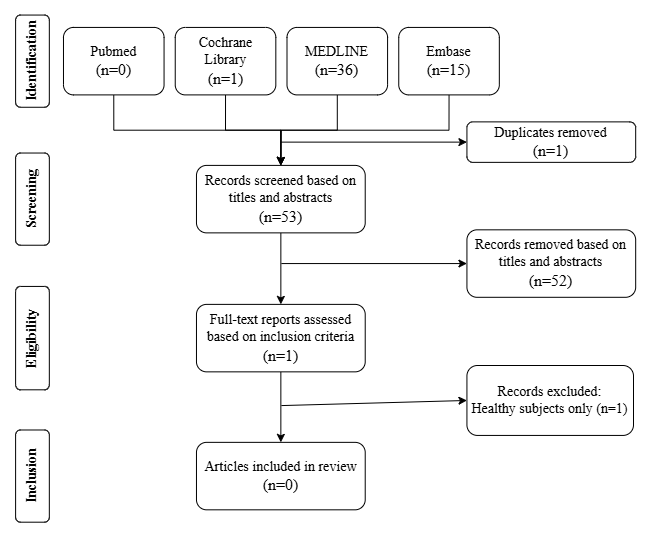
**

**Periodic Paralysis**

**
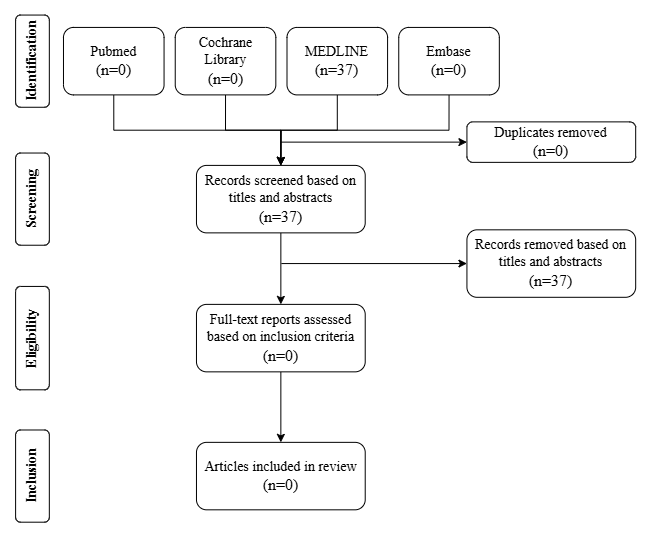
**
